# Aberrant right subclavian artery: case report and literature review

**DOI:** 10.1590/1677-5449.202101512

**Published:** 2023-02-20

**Authors:** Michel Nasser, Bruna Beatriz Petrocheli, Thais Keltke Santos Felippe, Beatriz Isola, Beatriz Caroline dos Santos Pereira, Ana Luiza Carvalho Sartoreli, João Marques Batista, Gustavo Muçouçah Sampaio Brandão

**Affiliations:** 1 Universidade Federal de São Carlos - UFSCar, Departamento de Medicina, São Carlos, SP, Brasil.

**Keywords:** aberrant right subclavian artery, vascular anomaly, Kommerell diverticulum, arteria lusoria

## Abstract

The aberrant right subclavian artery, also known as the arteria lusoria, is the most common aortic arch anomaly, occurring in 0.5 to 1% of the population. There is a higher prevalence in women and it is usually associated with other anatomical variations, such as the non-recurrent laryngeal nerve, present in 86.7% of cases. In the majority of cases, the aberrant right subclavian artery causes no symptoms. We describe this anomaly in an 82-year-old, hypertensive, and asymptomatic patient who had undergone a thoracoabdominal angiography to investigate a chronic DeBakey type III aortic dissection with dilation of the descending aorta. The aberrant right subclavian artery followed a retroesophageal course and was associated with a Kommerell diverticulum. In view of its rarity, we conducted an integrative bibliographic review of literature from the last 6 years indexed on the Medline, UpToDate, Lilacs, Scielo, and Portal Capes databases and discuss the most frequent anatomical changes, symptomatology, and therapeutic management adopted.

## INTRODUCTION

The aberrant right subclavian artery (ARSA), or arteria lusoria, is the most common aortic arch anomaly, occurring in 0.5 to 1% of the population.^1^^,^^2^ The name “lusoria” is derived from the term *dysphagia lusus naturae*,^3^ which can be loosely translated as “dysphagia caused by an aberration of nature” and was coined by Bayford to describe symptoms of dysphagia related to presence of an ARSA. Some recent studies^4^ have shown associations with chromosomopathies and cardiac and vascular malformations; but ARSA is an anatomic variant.

This variant is very often found by chance, since it is generally symptom free; but when it presents with dysphagia,^5^ coughing, chest pain, upper limb ischemia, and subclavian steal syndrome, the suspicion is that adjacent structures such as the esophagus and trachea are subjected to compression by the artery along its abnormal course or because of aneurysmal degeneration, known as Kommerell diverticulum.^6^^,^^7^^,^^8^^,^^9^^,^^10^^,^^11^

When present, and depending on its diameter, a Kommerell diverticulum can compress adjacent structures and if left undiagnosed may lead to rupture of the aneurysm, dissection of the aorta, or even recurrent pneumonia.^6^^,^^8^^,^^9^ There are also reports of rare cases of thrombosed Kommerell diverticula,^10^ with subclavian artery stenosis, which is a condition that can result in distal emboli and subclavian steal syndrome.

It is extremely important to diagnose Kommerell diverticulum, to enable monitoring and prevention of the main complications that may develop because of its presence.^6^^,^^8^

This project was approved by the Ethics Committee on January 27, 2022, under consolidated opinion number 5.213.980.

## CASE PRESENTATION

An 82-year-old, female, hypertensive, non-smoking, non-diabetic, multiparous, and asymptomatic patient was referred by outpatients in March of 2017 for investigation of a chronic DeBakey type III dissection associated with a dilatation of the descending aorta with a diameter of less than 5 centimeters, which had been identified by transesophageal Doppler ultrasonography. She did not complain of dysphagia, coughing, or right upper limb temperature changes and all of her pulses were normal.

**Figure 1:**
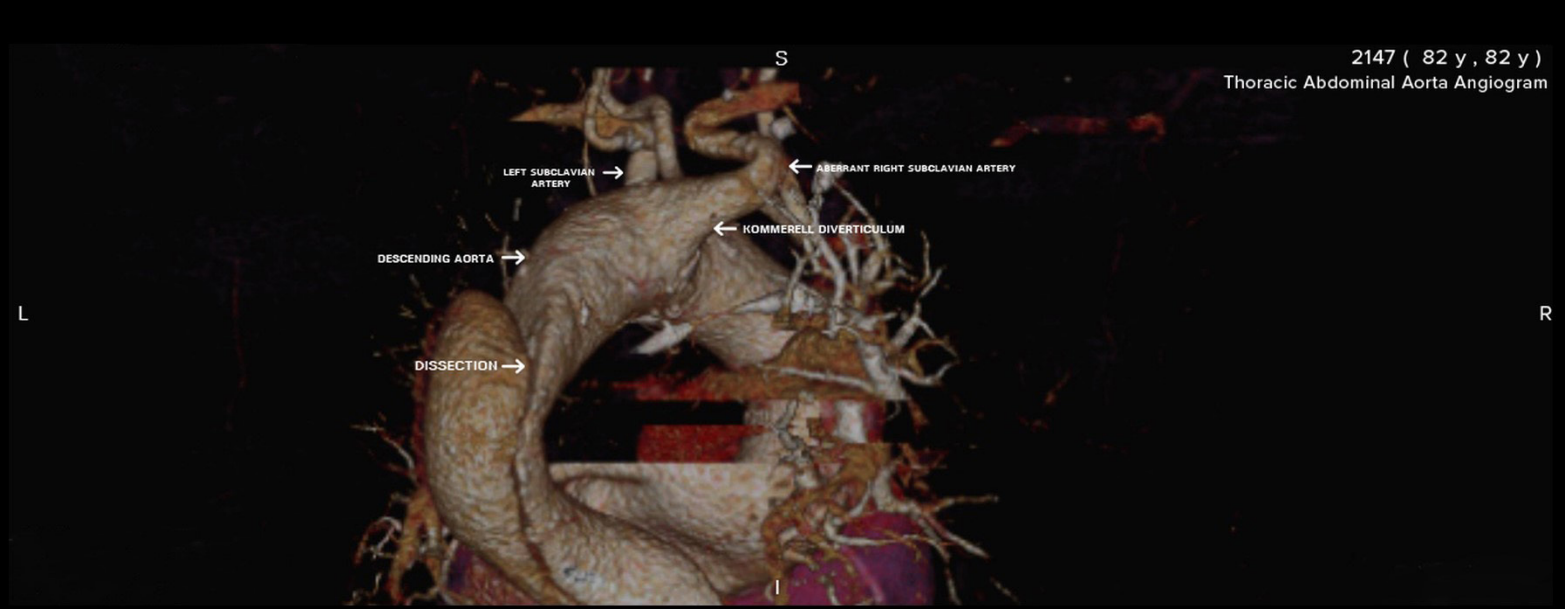
Posterolateral view of the aorta, showing the Kommerell diverticulum, the aberrant right subclavian artery, and dissection of the descending aorta.

During diagnostic confirmation by thoracoabdominal angiotomography, a variant arrangement of the aortic arch branches was observed: an ARSA was seen taking a retroesophageal path and with dilatation at the origin, characterizing a Kommerell diverticulum (Figure 1).

The patient was asymptomatic with respect to the ARSA, which is common in the majority of cases. The dissection was treated clinically, with prescriptions for beta blockers, antihypertensives, antiplatelet drugs, and a cholesterol reducing agent, plus continuous six-monthly follow-up appointments as a prophylactic measure. The patient died in August of 2021 from non-vascular causes.

## DISCUSSION

The aortic arch gives rise to three vessels: the brachiocephalic trunk (which divides into the right subclavian and right common carotid arteries), the left common carotid artery, and the left subclavian artery.^12^^,^^13^ These branches are classified as major elastic arteries and help to stabilize blood flow.

The subclavian arteries are formed during the embryonic period from the aortic arches, which emerge during the fourth week of intrauterine life. The right subclavian artery develops from the fourth right aortic arch, the dorsal aorta, and the seventh right intersegmental artery, while the left subclavian artery develops from the seventh left intersegmental artery.^14^

In rare cases, the primitive aortas and aortic arches may develop abnormally, leading to formation of an ARSA, which emerges when the right subclavian artery is formed by the distal portion of the right dorsal aorta and by the seventh intersegmental artery, while the fourth right aortic arch and the proximal part of the dorsal aorta, which would be involved in its formation, are obliterated. As a result, in the absence of the fourth right aortic arch, the aberrant right subclavian artery emerges as the last aortic branch.^15^

Along its course, an ARSA may cross the aortic arch transversely, posterior to the esophagus and the trachea in the direction of the right arm (in 80% of cases), anterior to the trachea (in 5% of cases), or even between the esophagus and the trachea (in 15% of cases), following a more linear path than the normal right subclavian artery.^16^^,^^17^^,^^18^^,^^19^

According to Molz and Burri,^20^ the incidence of an arteria lusoria is 58% in women and 42% in men. These data are in line with data reported by Polguj et al.,^21^ who identified a greater prevalence of ARSA in women (55.3%) than in men (44.7%). Jain et al.^22^ also found that an aberrant right subclavian artery is more prevalent in women.

There are also a number of possible anatomic variations in the origin and distribution of the main branches of the aortic arch when an aberrant right subclavian artery is present.

The Adachi and Willians classification divides ARSAs into four basic subtypes:^3^

**Figure 2:**
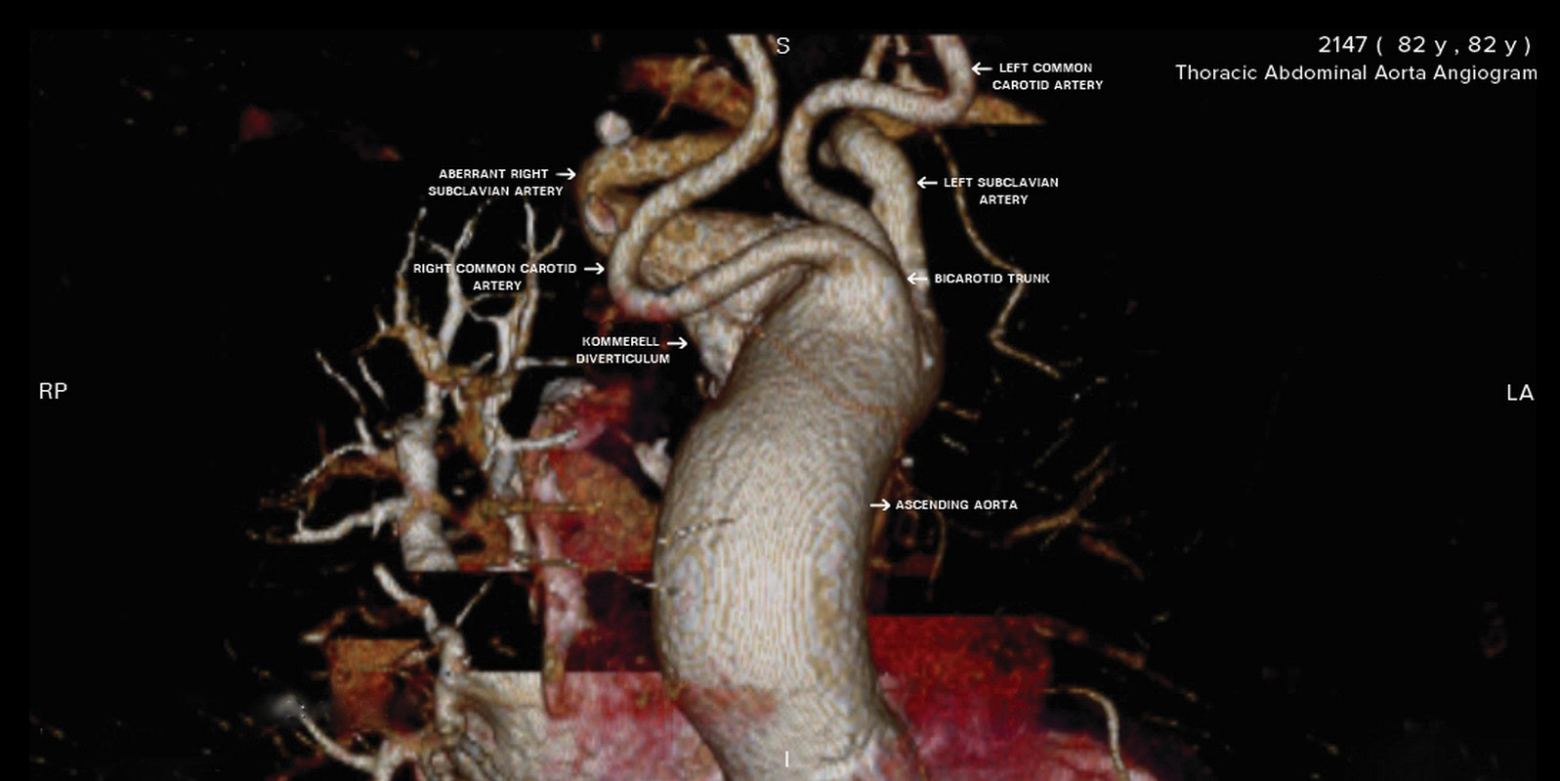
Anterior view of the aortic root, demonstrating a class H1 distribution, with a bicarotid trunk, a Kommerell diverticulum, and the aberrant right subclavian artery.
- Type N-1: in this type, distribution is the mirror-image of type G, since the aortic arch is on the right and a left subclavian artery imitates what would be an arteria lusoria.

- Type G-1: characterized by an ARSA originating from the distal portion of the aortic arch as its last branch. In this case, the remaining main branches follow the normal sequence (right common carotid artery, left common carotid artery, and left subclavian artery, all emerging directly from the aortic arch);
- Type CG-1: the ARSA is in the same anatomic position distal of the aortic arch as in type G, but the left vertebral artery originates directly from the aortic arch as an additional branch;
- Type H-1: the ARSA is also the last branch of the aortic arch, but in this case there is a bicarotid trunk, i.e., a single trunk that divides into two branches and gives rise to the right and left common carotid arteries, as seen in the case described here (Figure 2); and

Presence of an ARSA is commonly found in conjunction with other anatomic variations. One of these is a non-recurrent laryngeal nerve, found together with an ARSA in 86.7% of the studies analyzed.^23^ If its abnormal path is not identified in advance, it can result in surgical^24^^,^^25^ and post-surgical iatrogenic complications, such as paralysis of the vocal cords. In an integrative review of the MEDLINE, UpToDate, LILACS, SciELO, and Portal CAPES databases, we found 17 articles published during the last 6 years that report cases of patients with ARSA, in which clinical findings were symptomatic in 14 cases (82.4%) and asymptomatic in three cases (17.6%). Table 1 lists the treatments employed.

**Table 1:** Review of literature from the last 6 years indexed on MEDLINE, UpToDate, LILACS, SciELO, and the Portal CAPES reporting findings of an aberrant right subclavian artery (ARSA) combined with a Kommerell diverticulum.

**Study**	**Patient**	**Symptoms**	**Treatment and intervention**
Robles et al. (2019)^8^	Male, 8 years old, with moderate persistent asthma.	Dyspnea and wheezing	Clinical. Albuterol and systemic steroids because of shortness of breath. Outpatients follow-up for surgical intervention (left thoracotomy division of the vascular ring) to treat the Kommerell diverticulum (KD).
Onishi et al. (2020)^10^	Male, 81 years old. Acute pulmonary embolism, KD and thrombosed pulmonary arteries.	Dyspnea	Clinical. Treatment with rivaroxaban 30 mg/day anticoagulation. Computed tomography (CT) performed 7 days later showed reduction of thrombi in the pulmonary arteries and the KD.
Coşkun et al. (2019)^18^	Male, 67 years old. Smoker. Hypertension and chronic obstructive pulmonary disease (COPD), both under continuous pharmacological treatment.	Asymptomatic	Surgical. Surgical resection of the KD.
Domínguez-Massa et al. (2019)^6^	Male, 54 years old. Previously diagnosed ARSA.	Abrupt chest pain irradiating to the neck, dysphagia and dysphonia	Surgical. Resection of the aneurysmal segment.
Pessuti and Fontes (2019)^7^	Female, 76 years old.	Dysphagia, pain in the right hemithorax	Clinical.
Álvarez et al. (2020)^5^	Female, 18 years old.	Dysphagia	Clinical. Guidance on dietary changes and deglutition strategies. Referred to a cardiovascular surgeon for assessment and intervention was deferred.
Machado et al. (2016)^2^	Male, 56 years old.	Progressive claudication of the right upper limb and dizziness	Surgical. Endovascular.
Hanžič et al. (2019)^19^	Female, 69 years old.	Asymptomatic	Clinical. Guidance.
Araújo et al. (2015)^26^	Female, 70 years old. History of gastroesophageal reflux disease, treated with proton-pump inhibitors.	Occasional dysphagia	Clinical.
Xiong et al. (2020)^25^	Male, 53 years old. Hypertension and chronic renal failure.	Chest and back pain	Surgical. Endovascular repair.
Drullinsky et al. (2017)^27^	Female, 74 years old. Hypertension, osteoporosis, right hip joint replacement.	Weakness of the right upper limb with weakened pulses and fall in temperature	Surgical. Hybrid repair (endovascular and conventional surgery).
Suárez-Mantilla et al. (2018)^28^	Female, 3-month-old newborn.	Dysphagia, dry cough	Clinical. Surgery was recommended, but the patient’s family chose conservative treatment.
Bohatch et al. (2017)^29^	Female, 85 years old, hypertension.	Mild dysphagia for solids	Clinical. Watchful waiting and lifestyle changes.
Powell (2017)^30^	Female, 48 years old, healthy. Continuous treatment with proton-pump inhibitor for dysphagia of unknown causes.	Progressive dysphagia for solids with occasional regurgitations and worsening during the previous 2 years	Surgical. Extra-anatomic bypass of the aortic arch and right subclavian artery; endovascular repair 4 months later.
Thoreau et al. (2017)^31^	Female, 87 years old, without prior risk factors.	Subacute digital Ischemia of the phalanx of the right index finger, complicated by necrosis	Surgical. Ilomedin to control ischemia + surgery (supraclavicular cervicotomy) after finding KD + ARSA.
Marenchino and Domenech (2016)^32^	Male, 78 years old, hypertension, renal failure.	Dysphagia, back pain and severe malnutrition due to compression of the esophagus	Surgical. Extra-anatomic debranching and direct repair of the aorta, avoiding use OF deep hypothermic cardiac arrest and reducing cardiopulmonary bypass duration because of the comorbidities.
Faggioni et al. (2016)^33^	Female, 70 years old, pulmonary carcinoma diagnosed with cerebral metastases.	Asymptomatic	Clinical. Anticoagulation.

Treatment guidelines have not yet been established for Kommerell diverticula because of the small number of cases, but surgical procedures are indicated for symptomatic patients or those whose diverticula have cross-sectional diameters greater than or equal to 50 mm. For asymptomatic patients, some professionals recommend surgery to prevent complications, which can be performed using conventional, endovascular, or hybrid methods, but the decision must be made on a case-by-case basis, taking age and comorbidities into consideration.^6^^,^^9^

Clinical treatment of these patients aims to maintain heart rate below 60 beats per minute and systolic blood pressure between 100 and 120 mmHg, to reduce stress on the aorta wall and avoid progression of dissection. In combination with this, preventative treatment with cholesterol reducers and antiplatelet drugs has proven beneficial for primary and secondary prevention of thrombi-mediated events.

## CONCLUSIONS

An ARSA is a rare condition which in many cases does not provoke symptoms, meaning that is more often detected by chance during subsidiary examinations than clinically. It is the most common type of aortic arch variant and may pass anterior to the trachea, posterior to the esophagus, or between these two structures, and can also be found in conjunction with another variant, a non-recurrent laryngeal nerve, which is why it is extremely important to know about these different anatomic paths, especially for surgeons, to avoid damaging the structures involved.

Analysis of the recent literature indicates that surgical treatment should always be employed for symptomatic patients. Asymptomatic patients should be treated clinically and management should be founded on controlling hypertension and using platelet antiaggregants and cholesterol reducers as secondary prevention, in addition to continuous follow-up.
