# Supplementary material for: Aberrant right subclavian artery: case report and literature review
Source: J Vasc Bras. 2023 Feb 20;22:e20210151. doi: 10.1590/1677-5449.202101512 (PMC9968513; doi:10.1590/1677-5449.202101512)
Supplement: Translation

# Translation: Artéria subclávia direita aberrante: relato de caso e revisão de literatura

**DOI:** 10.1590/1677-5449.202101511

**Authors:** Michel Nasser, Bruna Beatriz Petrocheli, Thais Keltke Santos Felippe, Beatriz Isola, Beatriz Caroline dos Santos Pereira, Ana Luiza Carvalho Sartoreli, João Marques Batista, Gustavo Muçouçah Sampaio Brandão

**Affiliations:** 1 Universidade Federal de São Carlos - UFSCar, Departamento de Medicina, São Carlos, SP, Brasil.

**Keywords:** artéria subclávia direita aberrante, anomalia vascular, divertículo de Kommerell, artéria lusória

## Abstract

A artéria subclávia direita aberrante, também conhecida como artéria lusória, é a anomalia do arco aórtico mais comum, ocorrendo entre 0,5 e 1% da população. Possui prevalência em mulheres e normalmente está associada a outras variações anatômicas, como o nervo laríngeo não recorrente, presente em 86,7% dos casos. Em sua maioria, a artéria subclávia direita aberrante não apresenta sintomas. Descrevemos essa alteração em uma paciente de 82 anos, hipertensa e assintomática, que havia sido submetida a uma angiotomografia toracoabdominal para a avaliação de uma dissecção crônica tipo III (DeBakey) associada à dilatação de aorta descendente. No achado, a artéria subclávia direita aberrante apresentava percurso retroesofágico associado a um divertículo de Kommerell. Devido à raridade, realizamos revisão bibliográfica integrativa das bases de dados MEDLINE, UpToDate, LILACS, SciELO e Portal CAPES dos últimos 6 anos e discutimos as alterações anatômicas mais frequentes, a sintomatologia e as condutas terapêuticas adotadas.

## INTRODUÇÃO

A artéria subclávia direita aberrante (ASDA), ou artéria lusória, é a anomalia mais comum do arco aórtico, a qual ocorre entre 0,5 e 1% da população^1^^,^^2^. O nome “lusória” é derivado do termo *dysphagia lusus naturae*^3^ (em tradução livre, “disfagia causada por uma aberração da natureza”), cunhado por Bayford para descrever o sintoma de disfagia relacionado à presença da ASDA. Alguns estudos recentes^4^ têm mostrado sua associação com cromossomopatias e malformações cardíacas e vasculares; todavia, a ASDA é uma variante anatômica.

Sendo muitas vezes encontrada ao acaso, geralmente não apresenta sintomatologia; porém, perante a presença de disfagia^5^, tosse, dor torácica, isquemia de membros superiores e síndrome de roubo subclávio, suspeita-se de compressão de estruturas adjacentes, como esôfago e traqueia, pela artéria em seu percurso anormal ou devido à degeneração aneurismática, denominada divertículo de Kommerell^6^^,^^7^^,^^8^^,^^9^^,^^10^^,^^11^.

Quando presente, o divertículo de Kommerell, a depender de seu diâmetro, pode comprimir estruturas adjacentes e, se não diagnosticado, pode levar a uma ruptura do aneurisma, dissecção da aorta ou mesmo pneumonia recorrente^6^^,^^8^^,^^9^. Há ainda relatos de raros casos de divertículos de Kommerell trombosados^10^, com artéria subclávia estenosada, condição que pode resultar em quadros de embolia distal e síndrome do roubo subclávio.

O diagnóstico do divertículo de Kommerell é de suma importância, a fim de acompanhar e prevenir as principais complicações que podem surgir decorrentes de sua presença^6^^,^^8^.

O presente trabalho foi aprovado pelo Comitê de Ética no dia 27 de janeiro de 2022, tendo o seguinte número do parecer consubstanciado: 5.213.980.

## APRESENTAÇÃO DE CASO

Uma paciente do sexo feminino, com 82 anos de idade, hipertensa, não tabagista, não diabética, multípara e assintomática, foi encaminhada ambulatorialmente em março de 2017 para avaliação de uma dissecção crônica tipo III (DeBakey) associada à dilatação de aorta descendente menor que 5 centímetros de diâmetro, identificada por eco-Doppler transesofágico. A paciente não havia queixas de disfagia, tosse ou alterações de temperatura de membro superior direito, com todos os pulsos normais.

Na confirmação diagnóstica por angiotomografia toracoabdominal, foi identificada uma variante na disposição dos ramos do arco aórtico: uma ASDA notada pelo seu percurso retroesofágico associada a uma dilatação na origem, caracterizando, dessa forma, um divertículo de Kommerell (Figura 1).

**Figura 1:** Vista posterolateral da aorta, evidenciando o divertículo de Kommerell, a artéria subclávia direita aberrante e a dissecção da aorta descendente.

A paciente apresentava-se assintomática perante a presença da ASDA, como é frequente na maioria dos casos. O tratamento da dissecção se deu de forma clínica, apenas com prescrição de medicamentos betabloqueador, anti-hipertensivo, antiagregante plaquetário e redutor de colesterol, além de acompanhamento contínuo semestral como medida profilática. Em agosto de 2021, a paciente faleceu de causas não vasculares.

## DISCUSSÃO

O arco aórtico dá origem a três vasos: o tronco braquiocefálico (que se divide nas artérias subclávia direita e carótida comum direita), a artéria carótida comum esquerda e a artéria subclávia esquerda^12^^,^^13^. Esses ramos são classificados como grandes artérias elásticas e ajudam na estabilização do fluxo sanguíneo.

A formação das artérias subclávias durante o período embrionário ocorre a partir dos arcos aórticos que se originam na quarta semana da vida intrauterina, sendo que a artéria subclávia direita se desenvolve a partir do quarto arco aórtico direito, da aorta dorsal e da sétima artéria intersegmentar direita, enquanto a esquerda se desenvolve a partir da sétima artéria intersegmentar esquerda^14^.

Em casos raros, pode ocorrer o desenvolvimento anormal das aortas primitivas e dos arcos aórticos levando à formação da ASDA, a qual ocorre quando a artéria subclávia direita é formada pela porção distal da aorta dorsal direita e pela sétima artéria intersegmentar, enquanto o quarto arco aórtico direito e a parte proximal da aorta dorsal, que participariam de sua formação, são obliterados. Assim, na ausência do quarto arco aórtico direito, a artéria subclávia direita aberrante surge como último ramo aórtico^15^.

Em seu percurso, a ASDA pode cruzar transversalmente o arco aórtico, posteriormente ao esôfago e à traqueia em direção ao braço direito (em 80% dos casos), anteriormente à traqueia (em 5% dos casos) ou mesmo entre o esôfago e a traqueia (em 15% dos casos), em um trajeto mais linear do que a artéria subclávia direita normal^16^^,^^17^^,^^18^^,^^19^.

Segundo Molz e Burri20, a incidência da artéria lusória é de 58% em mulheres e 42% em homens. Esses dados estão em consonância aos obtidos por Polguj et al.^21^, os quais identificaram uma maior ocorrência da ASDA em mulheres (55,3%) do que em homens (44,7%). Jain et al.^22^ também constataram que a artéria subclávia direita aberrante é prevalente em mulheres.

Existem ainda algumas possíveis variações anatômicas na origem e distribuição dos ramos principais do arco aórtico na presença de uma artéria subclávia direita aberrante.

Segundo a classificação de Adachi e Willians, pode-se dividir a ASDA em quatro subtipos básicos^3^:

- Tipo G-1: se caracteriza pela origem da ASDA na porção distal do arco aórtico como o seu último ramo. Neste caso, os demais ramos principais seguem a sequência normal (artéria carótida comum direita, artéria carótida comum esquerda e artéria subclávia esquerda, todas partindo diretamente do arco aórtico);
- Tipo CG-1: tem a ASDA na mesma posição anatômica distal do arco aórtico que o tipo G, mas a artéria vertebral esquerda origina-se como um novo ramo direto do arco aórtico;
- Tipo H-1: possui igualmente a ASDA como último ramo do arco aórtico, mas neste caso com a presença de um tronco bicarotídeo, isto é, um único tronco que se divide em dois ramos e dá origem às artérias carótidas comuns direita e esquerda, como visualizado no caso em estudo (Figura 2); e

**Figura 2:** Vista anterior da raiz aórtica, evidenciando a classificação do tipo H1 com o tronco bicarotídeo, o divertículo de Kommerell e a artéria subclávia direita aberrante.
- Tipo N-1: sua distribuição caracteriza-se como uma imagem enantiomorfa do tipo G, pois apresenta um arco aórtico à direita com uma artéria subclávia esquerda imitando o que seria a artéria lusória.

A presença de uma ASDA é um achado em comum a outras variações anatômicas. Uma delas é o nervo laríngeo não recorrente, encontrado em conjunto com a ASDA em 86,7% dos estudos analisados^23^ e cujo trajeto incomum, não identificado previamente, pode levar a complicações iatrogênicas cirúrgicas^24^^,^^25^ e pós-cirúrgicas, como a paralisia das cordas vocais. Em revisão integrativa nas bases de dados MEDLINE, UpToDate, LILACS, SciELO e Portal CAPES, encontramos 17 artigos dos últimos 6 anos que relatam casos de pacientes com ASDA, dos quais os achados clínicos eram sintomáticos em 14 casos (82,4%) e assintomáticos em três (17,6%). Os tratamentos executados foram realizados conforme a Tabela 1.

**Tabela 1:** Revisão nas bases de dados MEDLINE, UpToDate, LILACS, SciELO e Portal CAPES de achados de artéria subclávia direita aberrante (ASDA) associados a divertículo de Kommerell dos últimos 6 anos.

**Estudo**	**Paciente**	**Sintomas**	**Tratamento e intervenção**
Robles et al. (2019)^8^	Masculino, 8 anos. Quadro de asma moderada persistente.	Dispneia e respiração ofegante	Clínico. Albuterol e esteroides sistêmicos devido a falta de ar. Acompanhamento ambulatorial para intervenção cirúrgica (divisão de toracotomia esquerda do anel vascular) do divertículo de Kommerell (DK).
Onishi et al. (2020)^10^	Masculino, 81 anos. Quadro agudo de embolismo pulmonar, DK e artérias pulmonares trombosadas.	Dispneia	Clínico. Tratamento com anticoagulante rivaroxaban 30 mg/dia. Tomografia computadorizada (TC) realizada 7 dias depois mostrou redução dos trombos nas artérias pulmonares e no DK.
Coşkun et al. (2019)^18^	Masculino, 67 anos. Fumante. Hipertensão e doença pulmonar obstrutiva crônica (DPOC), ambos em tratamento medicamentoso contínuo.	Assintomático	Cirúrgico. Ressecção cirúrgica do DK.
Domínguez-Massa et al. (2019)^6^	Masculino, 54 anos. ASDA previamente diagnosticada.	Dor torácica abrupta irradiando para o pescoço, disfagia e disfonia	Cirúrgico. Ressecção do segmento aneurismático.
Pessuti e Fontes (2019)^7^	Feminino, 76 anos.	Disfagia, dor no hemitórax direito	Clínico.
Álvarez et al. (2020)^5^	Feminino, 18 anos.	Disfagia	Clínico. Orientações sobre modificações dietéticas e estratégias de deglutição. Encaminhada e avaliada por cirurgião cardiovascular, e a intervenção foi deferida.
Machado et al. (2016)^2^	Masculino, 56 anos.	Claudicação progressiva do membro superior direito associada a tonturas	Cirúrgico. Endovascular.
Hanžič et al. (2019)^19^	Feminino, 69 anos.	Assintomático	Clínico. Orientações.
Araújo et al. (2015)^26^	Feminino, 70 anos. Histórico de doença do refluxo gastroesofágico, tratada com bomba inibidor de prótons.	Disfagia ocasional	Clínico.
Xiong et al. (2020)^25^	Masculino, 53 anos. Hipertensão e insuficiência renal crônica.	Dor torácica e nas costas	Cirúrgico. Reparo endovascular.
Drullinsky et al. (2017)^27^	Feminino, 74 anos. Hipertensão, osteoporose, artroplastia do quadril direito.	Fraqueza no membro superior direito com diminuição de pulso e temperatura	Cirúrgico. Reparo híbrido (endovascular e cirúrgico convencional).
Suárez-Mantilla et al. (2018)^28^	Feminino, recém-nascida de 3 meses de idade.	Disfagia, tosse seca	Clínico. Aconselharam cirurgia, mas família optou por tratamento conservador.
Bohatch et al. (2017)^29^	Feminino, 85 anos, hipertensão.	Disfagia leve para sólidos	Clínico. Tratado de forma expectante com modificação do estilo de vida.
Powell (2017)^30^	Feminino, 48 anos, saudável. Tratamento contínuo com inibidor de bomba de prótons para disfagia de causa desconhecida.	Disfagia progressiva para sólidos com eventuais regurgitações e piora nos últimos 2 anos	Cirúrgico. *Bypass* extra-anatômico do arco aórtico e da artéria subclávia direita; 4 meses depois, reparo endovascular.
Thoreau et al. (2017)^31^	Feminino, 87 anos, sem fatores de risco antecedentes.	Isquemia digital subaguda da falange do dedo indicador direito, complicada por necrose	Cirúrgico. Ilomedina para conter a isquemia + cirurgia (cervicotomia supraclavicular) após achado de DK + ASDA.
Marenchino e Domenech (2016)^32^	Masculino, 78 anos, hipertensão, insuficiência renal.	Disfagia, dor torácica e desnutrição severa devido a compressão esofágica	Cirúrgico. Desramificação extra-anatômica e reparo direto da aorta, evitando utilizar hipotermia profunda com parada circulatória total (DHCA, de *deep hypothermic cardiac arrest*) e diminuindo o tempo de *bypass* cardiopulmonar devido às comorbidades.
Faggioni et al. (2016)^33^	Feminino, 70 anos, carcinoma pulmonar diagnosticado com metástases cerebrais.	Assintomático	Clínico. Anticoagulante.

Ainda não existem diretrizes de tratamento estabelecidas para o divertículo de Kommerell devido à pequena quantidade de casos existentes, porém procedimentos cirúrgicos são indicados para pacientes sintomáticos ou com divertículos de diâmetro transversal maior ou igual do que 50 mm. Para pacientes assintomáticos, a cirurgia, que pode ser convencional, endovascular ou híbrida, é recomendada por alguns profissionais como prevenção de complicações, mas a escolha é feita caso a caso, levando em consideração a idade e as comorbidades^6^^,^^9^.

O tratamento clínico do paciente consiste em manter a frequência cardíaca menor que 60 batimentos por minuto e a pressão arterial sistólica entre 100 e 120 mmHg, a fim de reduzir o estresse na parede aórtica e evitar a progressão de uma dissecção. Associado a isso, o tratamento preventivo com redutor de colesterol e antiagregante plaquetário tem sido benéfico na prevenção primária e secundária de eventos mediados por trombos.

## CONCLUSÕES

A ASDA é uma condição rara que, em muitos casos, não apresenta sintomas, o que a faz ser mais comumente descoberta ao acaso através de exames subsidiários do que de maneira clínica. Ela representa a variação mais comum do arco aórtico, podendo ser anterior à traqueia, posterior ao esôfago ou cruzar entre esses órgãos, e ainda pode vir associada a outra variação, que é o nervo laríngeo não recorrente, assim confirmando a extrema importância do conhecimento desses diferentes trajetos anatômicos, especialmente por cirurgiões, a fim de evitar lesões dessas estruturas.

Conforme a análise da literatura recente, o tratamento cirúrgico deve ser efetuado sempre em pacientes sintomáticos. Os assintomáticos devem ser tratados clinicamente, e sua base deve ser o controle de hipertensão, antiagregantes plaquetários e redutores de colesterol como prevenção secundária, além de acompanhamento contínuo.
